# Top of the podium, at what cost? injuries in female international elite climbers

**DOI:** 10.3389/fspor.2023.1121831

**Published:** 2023-06-16

**Authors:** G. Grønhaug, L. M. Joubert, A. H. Saeterbakken, S. N. Drum, M. C. Nelson

**Affiliations:** ^1^ Department of Sport, Food and Natural Sciences, Faculty of Education, Arts and Sports, Western Norway University of Applied Sciences, Campus, Sogndal, SFC, United States; ^2^ School of Health and Human Performance, Northern Michigan University, Marquette, MI, United States; ^3^ Student-Athlete High Performance Center and Sims-Treharne Collaborative Research Laboratory; Department of Health Sciences, Northern Arizona University, Flagstaff, AZ, United States; ^4^ School of Health and Human Performance, Northern Michigan University, Marquette, MI, United States

**Keywords:** climbing, climbing injuries, BMI, eating disorders, sports injuries, sport medicine

## Abstract

**Objectives:**

Competitive sport climbing has made its way to the Olympic stage. This prestige has brought about route setting and training alterations which presumably affect injury epidemiology. Most of the climbing injury literature contains male climbers and lacks high performing athletes. Studies with both female and male climbers, rarely included separate analyses for performance level or sex. Therefore, injury concerns for elite female competitive climbers are impossible to discern. A former study examined the prevalence of amenorrhea in elite international female climbers (*n* = 114) and reported that 53.5% had at least one injury in the past 12 months, but injury details were excluded. This study's aim was to report these injury details and their associations with BMI, menstrual status and eating disorders of the cohort.

**Methods:**

Online survey was emailed to competitive female climbers recruited through the IFSC database between June and August 2021. Data was analyzed using Mann–Whitney *U*, *χ*^2^ and logistic regression.

**Results:**

229 registered IFSC climbers opened the questionnaire and 114 (49.7%) provided valid responses. Respondents (mean ± SD; age = 22.9 ± 5 year) represented 30 different countries and more than half (53.5%, *n* = 61) reported an injury in the prior 12 months with the majority in shoulders (37.7%, *n* = 23) and fingers (34.4%, *n* = 21). Injury prevalence in climbers with amenorrhea was 55.6% (*n* = 10). BMI was not a significant predictor of injury risk (OR = 1.082, 95% CI: 0.89, 1.3; *p* = 0.440) while accounting for current ED over the past 12 months. However, the odds ratio for having an injury was doubled for those with an ED (OR = 2.129, 95% CI: 0.905, 5.010; *p* = 0.08).

**Conclusion:**

With over half reporting recent injuries (<12 months) mostly to shoulders and fingers, development of new strategies for injury prevention in competitive female climbers are warranted. In addition, climbers with disordered eating behaviors and/or menstrual disturbances might be more prone to injury. More research in this population is required. Suitable screening to prevent these health issues and proper monitoring of these athletes are paramount to long-term athlete success.

## 1. Introduction

Climbing has gained momentum as a competitive sport, especially with its recent debut in the 2020 Tokyo Olympic Games. With the required Olympic climbing format, which currently (Paris 2024) includes two medals for each sex: one medal for a combination of boulder + lead (combined) and one for speed, it's expected that more task-specific training strategies will be employed (1–5). It is likely that these objectives will create higher injury susceptibility to these competitive climbers. Additionally, climbers with lower abilities and route setters at commercial gyms tend to look to the elite for inspiration. Thus, the anticipated augmented volume-overload training in elite climbers may create higher injury susceptibility at the pre-Olympic level and most likely will impact injury rates in climbers of all abilities.

In general, chronic injuries in sports result from repetitive movement with high stress including either excessive loading, insufficient recovery and/or inadequate energy intake (6–10). Previous studies have demonstrated that fingers, elbows, and shoulders are the most prevalent site of injury in climbing (11–14). Most studies examining injury rates of climbers that include females and males do not report their injury rates or injury sites between these two sexes (15–21). Climbing style (i.e., top rope, bouldering, speed) (22, 23), performance level (i.e., beginner, advanced, elite) and competitiveness (local gym, national, international) were also not often distinguished within the climbing injury literature (15–21). Lutter et al. (24) presented a narrative review of the literature on injuries in competition climbing and concluded that data was scarce, of low quality and only one study included a sex-specific analysis (25). Furthermore, the majority of data collected on competitive climbers has been dominated by male participants ranging from 60%–100% (24). One study that collected injury survey data on athletes (312 females, 262 males) competing in a variety of sports found sex differences occurred for injury location as well as in type of sport and were somewhat explained by the differences in training hours (26). Therefore, more research is needed on female climbers, especially in high-level competitors and their rates of injury, location and their etiology.

Thus, the aim of this paper was to report the injury data collected in a previously published study that examined the prevalence of amenorrhea in elite-level female climbers registered with the International Federation of Sport Climbing (IFSC) (27). Additionally, we conducted an exploratory analysis to understand whether factors such as body mass index (BMI), eating disorders (ED) or menstrual status were associated with the number of injuries in this cohort.

## 2. Methods

An electronic survey was developed in Qualtrics (Qualtrics XM 2021, Provo UT) and consisted of a total of 33 questions. The survey was distributed by the IFSC to competition climbers with an international license. A total of 229 climbers registered in the IFSC database opened the questionnaire and 114 participants (49.7%) completed the questions in full. The survey included questions developed by the researchers related to the following sections: (1) demographics and anthropometrics (e.g., height, weight), (2) climbing resume (e.g., training volume, discipline), (3) behaviors related to changing body weight, (4) eating behaviors, (5) injuries and (6) menstrual history. Questions were formatted for responses that were multiple choice (i.e., select one answer and select all that apply), sliding scale, and text-entry/open-ended. Injury-related questions asked about the number of injuries and location of injury (e.g., finger, arm, calf). Participants also self-reported whether they thought their injury was classified as acute (e.g., an injury with a sudden onset) or chronic/overuse (e.g., an on-going issue).

Climbers were recruited through the IFSC database between June and August 2021. All climbers were required to be licensed with one of the 57 IFSC federations (28, 3) if competing in IFSC sanctioned events prior to a competition for the calendar year. Survey links were dispersed to all IFSC licensed members via email with consent collected after opening the survey and advancing to the second page. Due to this recruitment process, the researchers were unable to track how many female athletes received this email and/or opened it. The major outcome variable of the study, injury prevalence, was defined as the number of participants that responded yes to having at least one injury within the past 12 months. Factors hypothesized to influence injury rate that were explored included: body mass index (BMI; kg/m^2^), menstrual status (i.e., amenorrhea, no amenorrhea), and eating disorder prominence (i.e., eating disorder, no eating disorder).

BMI was classified as followed: <18.5 kg/m^2^, underweight; 18.5–24.9 kg/m^2^, healthy; 25.0–29.9 kg/m^2^, overweight, and ≥30.0 kg/m^2^, obese.

Eating disorder status (i.e., eating disorder, no eating disorder) was determined according to how respondents replied to prompt 31 on the survey. A “no eating disorder” classification was assigned if any of these 3 were checked “I have disordered eating patterns”, “I don’t have any of the above issues currently” or “I am unsure”. Further detail on the methods has been previously reported (29).

### 2.1. Statistics

For descriptive statistics, categorical data is reported as *n* (%) and continuous data is reported as mean ± standard deviation.

For exploratory analyses, a Mann-Whitney U independent samples test was used to determine whether there was a statistical difference between the overall number of self-reported injuries between climbers meeting criteria for amenorrhea compared to non-amenorrhea. Effect size for the Mann–Whitney *U* tests was calculated as *r* = *z*/√*n* and defined as: small effect, *r* = .1; medium effect, *r* = .3, and large effect, *r* = .5 (29).

A *χ*^2^ test was used to determine if there was a statistical difference between the prevalence of injury within the past 12 months between climbers who met criteria for amenorrhea compared to those who did not meet criteria for amenorrhea. Lastly, a logistic regression was used to assess whether BMI was associated with higher odds of self-reported injury (0 = no injury, 1 = injury), accounting for eating disorder status (0 = no eating disorder, 1 = eating disorder) in the model. Results were computed using IBM SPSS Statistics for Windows, version 28.0 (IBM Corp., Armonk, N.Y., USA). Statistical significance was defined as *p* < 0.05.

## 3. Results

### 3.1. Demographic and training variables

On average, the sample of 114 respondents included female climbers from 30 different countries, were aged 22.9 ± 5 years spanning from 16 to 40 years. Participants reported partaking in their first competition at the age of 12.9 ± 5.1 years and ranged from 6 to 30 years. The average BMI (range 15.4–27.2) of the participants (*n* = 110) was classified as healthy at 20.7 ± 1.9 kg/m^2^. Three climbers did not report their height and one climber did not report weight, thus BMI could not be calculated for 4 climbers. A total of 18 climbers (15.8%) were identified as meeting criteria for amenorrhea and 37 (32.4%) indicated they had at least one eating disorder. Of the 18 climbers classified with amenorrhea 10 (55.6%) reported at least one injury the past 12 months.

The respondents reported training for an average of 3.4 ± 1.2 h per training day and 5.2 ± 1.7 days per week (9 athletes reported that they were not currently training) with the majority of climbers (82; 78%) training at least 5 days per week. Of those training, 20 (17.5%) athletes had double trainings one day per week, 31 (27.2%) had double trainings two days per week, 11 (9.2%) had double trainings three days per week, 8 (7.0%) had double trainings four days per week, and 3 (2.6%) indicated that five days a week they had double training sessions within the same day. In the past 6 months, 17 (14.9%) competed in speed, 60 (52.6%) competed in bouldering, and 40 (35.1%) in lead. Only 11 (10%) had competed in a combined event (scores tallied in two or more disciplines within the same competition). Almost half of the athletes, 51 (45%) received financial sponsorship for sport climbing within the past 6 months.

### 3.2. Self-reported injury prevalence

Of the respondents, 61 (53.5%) reported they had experienced at least one injury in the past 12 months. The majority of the injured climbers experienced only one (21.9%, *n* = 25) or two (21.9%, *n* = 25) injuries. Only a few climbers reported three injuries (7%, *n* = 8) or four (2.6%, *n* = 3) injuries and there were no climbers who reported five or more injuries within the past 12 months. The majority of the injuries were to the shoulder (37.7%, *n* = 23) and finger(s) (34.4%, *n* = 21) followed by ankle/foot (32.8%, *n* = 20) and knee (27.9%, *n* = 17). All athletes who reported an injury stated that they sought a health professional for treatment for the injury.

### 3.3. Exploratory analyses of factors associated with self-reported injury

The prevalence of injury in those who met criteria for amenorrhea was 55.6% (*n* = 10). On average, there was no difference (*U* = 241.5, *z* = −0.284, *p* = 0.78, *r* = −0.04) in the number of injuries self-reported by climbers who met criteria for amenorrhea (*n* = 10, 1.70 ± 0.68, median = 2) compared to those who did not meet criteria for amenorrhea (*n* = 51, 1.84 ± 0.88, median = 2). The proportion of climbers self-reporting an injury within the last 12 months who met criteria for amenorrhea was not different between the climbers who did not meet criteria for amenorrhea [Chi square (*χ*^2^) = 0.036, *p* = 0.85]. Body mass index was not a significant predictor of injury risk (OR = 1.082, 95% CI: 0.89, 1.3; *p* = 0.440) while accounting for current eating disorders over the past 12 months. Although not statistically significant, the odds ratio for having an injury was doubled for participants indicating they had an eating disorder (OR = 2.129, 95% CI: 0.905, 5.010; *p* = 0.08).

## 4. Discussion

To the best of our knowledge the present study is the first to focus on injuries among international elite competitive female climbers. The main finding was that 53.5% of the athletes reported at least one injury within the past year, mostly injured shoulders and fingers. Compared to other individual sports, the rate of injuries in climbers, whether chronic or acute, is almost twice as high (30). Additionally, in many sports, elite athletes experience more injuries than lower-level athletes, and individual sports have fewer injuries than team sports (31). Although the findings of the present study with elite-level female climbers presents an injury rate greater than what is expected when comparing with other sports (31), it is still in line with a previous study of elite competitive climbers (32). Furthermore, compared with female artistic gymnasts the rate of shoulder injuries were similar (33) suggesting that the load to the shoulders in climbing is comparable to gymnastics. Comparing the present study with previous studies is difficult as most do not report sex-specific analyses. Still, the rate of injuries has been more or less the same for more than a decade and the one previous study that reported sex-specific analyses found similar injury rates in elite female climbers (13, 34).

### 4.1. Injuries

Injuries among climbers at a high level of performance has been assessed in 11 studies (24) reporting injury rates from 50% (13) to 61% (34). Thus, the present findings are in line with or slightly lower than previous studies. Still, comparisons between studies is challenging due to differences in the populations studied and the difference in the definition of an “elite” or “competitive” athlete. In the present study we included only those who were registered as competing in international level events organized or recognized by the IFSC. Thus, the present study is the first to present analyses on injuries in an elite group of female climbers.

Lutter et al. (24) speculated that a change in the onset of injury site may come as a consequence of new route setting techniques and/or sizes of holds or volumes on indoor walls and competitive settings (24). If such a change is to come it is likely to occur first among the high performing athletes who initiate new trends in route setting and training. Route setting on climbing walls and in competitions are to climbing what course setting is to ski racing. The ski race course design influences skier injuries in ski racing, which has been widely debated and studied for several years (35, 36). These reports have guided stakeholders to minimize the risk for injuries by changing the ski course and by developing injury prevention training programs for both the elite and recreational athletes (37). Minimizing injury risks by analyzing and interpreting movement patterns and setting climbing routes accordingly might help climbers like it has helped skiers.

Previous studies on injuries in climbing have found the fingers to be injured more often than any other anatomical site (18, 25, 34, 15, 17). The present study is the first to show more injuries to the shoulders than the fingers (Table 1) in a climbing population. When compared to the only other sex-specific analysis from 2018 (34) there is a change from fingers being most prevalent in the previous study (29.8% fingers vs. 21.9% shoulders) to shoulders in the present study (38% shoulders vs. 34% fingers).

**Table 1:** Number of injuries (*n* = 132) self-reported in the IFSC female athletes who experienced an injury (*n* = 61) during the year prior to June-August 2021.

Injury site	Shoulder	Finger	Foot/ankle	Knee	Elbow	Low back	Wrist	Hip	Neck	Thigh	Toe	Head
Acute	11	11	16	8	5	5	1	3	2	1	2	0
Chronic	12	10	4	9	10	9	6	3	2	1	0	1
Total number of injuries	23	21	20	17	15	14	7	6	4	2	2	1

This possible change in the most prevalent site of injuries for the female climber from the fingers to the shoulders might be a sign of how the change in route setting has evolved over the last decade. Climbing routes have transformed from being slightly overhanging walls to severely overhung that involve several no-feet jumps. However, it must be noted that the difference in number of injuries in the present study between the shoulders and fingers were small (23 vs. 21 cases or 38% vs. 34%). Further studies are needed before we can conclude if this is a matter of the present study focusing on elite level female climbers or if the findings in this study are the first to document a shift in the epidemiology of injuries in climbing.

The findings in the present study showed a higher prevalence of injuries to the knees and ankles than in one other study including injuries of female climbers (34). Combined with the shoulders as the most prevalent site of injury in climbing in the present study, the finding of more injuries to the knees further strengthens the anticipation that there is a shift in terms of where and how often climbers are injured.

Most chronic injuries may be prevented with appropriate action by athletes, stakeholders and organizers of the sport. Similar to competitive skiing, some climbing injuries might be prevented by adjusting training and resting protocols, and/or changing the competition rules or routes.

Regardless whether the current study premiers a shift in injury site or not, more focus is clearly needed on climbing shoulder injuries in terms of potential diagnosis, treatment and return to sport protocols.

### 4.2. BMI/injuries

There is a dearth of research looking at possible interaction between BMI and injuries in climbers. One previous study (38) used an univariable general linear model to assess a potential association of higher BMI and injuries and found none. In the present study, BMI was not a significant predictor of injury risk (OR = 1.082, 95% CI: 0.89, 1.3; *p* = 0.440) while accounting for current eating disorders over the past 12 months. Although not statistically significant, the odds ratio for having an injury was doubled for participants indicating they had an eating disorder (OR = 2.129, 95% CI: 0.905, 5.010; *p* = 0.08). Still with a 2.1 odds ratio, it raises awareness and skepticism to the practical use of BMI as a tool for health monitoring in climbing. This supports the conclusion of Joubert et al. (39) that there is a need for better health monitoring for athletes and inclusion of education for both trainers and athletes to avoid injuries related to having a low BMI, eating disorders or disordered eating behaviors. While not all cases of low BMI are a result of low energy availability, any climber may be in an energy deficit at any given time. If that time of energy deficit coincides with high loads of training or an injury, recovery will most likely take longer than when compared with recovery with adequate dietary energy intake.

### 4.3. Medical aid

All of the climbers in the present study who reported an injury sought medical aid (Table 2) from a health professional. This is contradictory to the other studies that assessed the use of health care among injured climbers (40, 41). Grønhaug & Saeterbakken (41) reported that although the majority of climbers did not seek health care, the female climbers were more likely to seek medical aid than their male counterparts (41.7% vs. 27.3%).

**Table 2:** Overview of injuries and use of health care.

	Yes		No	
	*n*	%	*n*	%
Injury past 12 months	61	53.5	53	46.5
**Number of injuries**				
1	25	21.9		
2	25	21.9		
3	8	7		
4	3	2.6		
**Did you seek health care?**				
	61	100		

As all the respondents in the present study sought medical aid, it is most likely an accessibility benefit to the climbers in the present study, since each team within the IFSC is required to have a medical commission. Additionally, the use of health care might be more about whether or not the climbers believe that the health personnel is capable to help with their injury. The female international elite climbers are all part of national teams within their own federations accompanied by health personnel that presumably are knowledgeable to treat climbing specific injuries.

### 4.4. Strengths and limitations

This study was a cross-sectional open on-line survey where injury prevalence was only a portion of the questions asked. It is very likely that some IFSC registered climbers with injuries may have been reluctant to take part in an online survey that included questions about menstrual health and eating behaviors.

The survey was open during the three months while most participants were in the midst of the international competitive season. This timing may have influenced their responses on questions regarding body weight. This may have been a prime time for these athletes to lower their weight to competition weight and the self-reported data collected may not have reflected their usual body weight.

A weakness of the study was absence of a medical examination on the reported injuries. Although the questionnaire specified that the study inquired about injuries, it is not guaranteed that the respondents reported the correct number of injuries within the past 12 months, or simply reported a sensation of pain that may or may not have been a true injury. Also, the survey neglected to ask if the injury occurred during climbing. Still, this is a weakness of all self-reported studies on the prevalence of injuries in sports.

A strength of the study was that all participants were international elite climbers. Performing research on a specific group of athletes makes the results easier to interpret and thereby increases the likelihood that the results may be used to develop medical screening guidelines, education and injury prevention strategies specifically targeted to this population.

## 5. Conclusion

With our cohort majority reporting injuries (<12 months) mostly to shoulders and fingers, this calls for development of new strategies for injury prevention to reduce injury susceptibility in female climbers. In addition, although this research did not make a strong case for this, climbers with disordered eating behaviors and/or menstrual disturbances might be more prone to injuries and require medical care interventions to attenuate injuries and protect health. More research on female competitive climbers is clearly needed. Health monitoring and injury prevention are paramount to long-term athlete success in other sports and climbing should be no exception.

## Data Availability

The raw data supporting the conclusions of this article will be made available by the authors, without undue reservation.

## Supplementary material

Supplementary Table 1 Full overview of survey questions.
